# The biological microprocessor, or how to build a computer with biological parts

**DOI:** 10.5936/csbj.201304003

**Published:** 2013-06-26

**Authors:** Gerd HG Moe-Behrens

**Affiliations:** a Leukippos Institute, Berlin, Germany

## Abstract

Systemics, a revolutionary paradigm shift in scientific thinking, with applications in systems biology, and synthetic biology, have led to the idea of using silicon computers and their engineering principles as a blueprint for the engineering of a similar machine made from biological parts. Here we describe these building blocks and how they can be assembled to a general purpose computer system, a biological microprocessor. Such a system consists of biological parts building an input / output device, an arithmetic logic unit, a control unit, memory, and wires (busses) to interconnect these components. A biocomputer can be used to monitor and control a biological system.

## Introduction

Nature and computers are words that used to mean unrelated things. However, this view changed, starting in the 1940s, when a revolutionary scientific paradigm, systemics based on platonic idealistic philosophy, gained popularity [1] [2] [3].

The roots of philosophical idealism based systemics goes back to Plato. A centerpiece of Plato's (428/7 to 348/7 BC) work is his theory of forms, also called theory of ideas [2]. Forms are archetypes, blueprints, the essences of the various phenomena of the same thing. The superior world consists, due to Plato, of mathematical objects, terms and non-materialistic abstract ideas. Moreover, Plato introduced in his dialogue Philebus a concept called System [4]. A system is according to Plato a model for thinking about how complex structures are developed. Another idealistic philosopher, Kant, introduced, in 1790, in his Critique of Judgment the concept of self-organizing [5]. Idealistic concepts based systemics have become important in contemporary science in order to understand complexity and big data problems. Between the 1950s and 60s three groundbreaking works were published: 1948, Norbert Wiener publishes “Cybernetics or Control and communication in the animal and machine” [1]. In 1955 William Ross Ashby's “Introduction to cybernetics” came out [6]. 1968, Ludwig Bertalanffy published “General System theory: Foundations, Development, Applications” [7]. Bertalanaffy defined the concept of systems. Cybernetics explains complex systems that exist of a large number of interacting and interrelated parts. Wiener and Ashby pioneered the use of mathematics to study systems. This systems theory was further developed in the following years. Important contributions to the field are by Heinz Foerster, whose work focused on cybernetics, the exploration of regulatory systems, and who founded in 1958 the Biological Computer Lab (BCL) at the Department of Electrical Engineering at the University of Illinois [8]. The work of the BCL was focused on the similarities in cybernetic systems and electronics and especially biology inspired computing [9]. Other important contributions to systemics are by the Nobel-prize winning work of Ilya Prigogine on self-organization and his systems theory concepts in thermodynamics [10]. Furthermore: Mitchell Feigenbaums work on Chaos theory [11]. Contemporary application finds systems theory in bioscience in fields such as systems biology, and its practical application synthetic biology [12]. The term systems biology was created by Bertalanffy in 1928 [13]. Systems biology focuses on complex interactions in biological systems by applying a holistic perspective [12].

Altogether, this kind of thinking has led to the identification of ideas behind data processing in nature, but also in machines, such as silicon computers.

## Natural Computing

**Figure 1:**
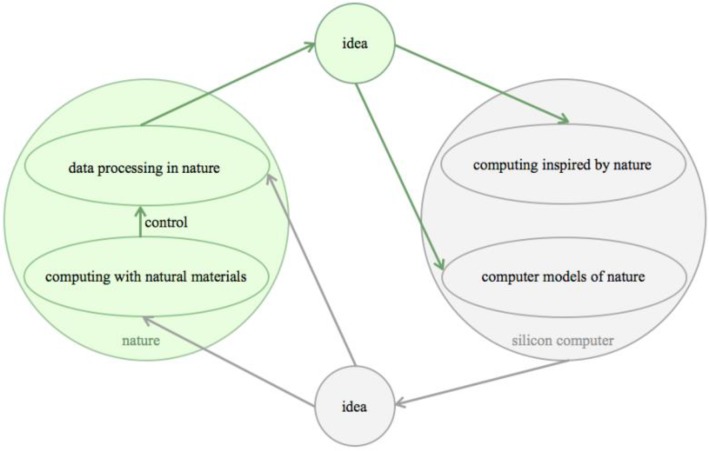
Natural computing: A platonic idea is an archetype, a blueprint, the essence of various phenomena of the same thing. Systemics and systems biology are such ideas, describing data processing systems in nature in terms of mathematics and formal logic. Systemic ideas have been used as a blueprint for silicon computing. Ideas derived from the observation of nature have also inspired computer models of nature. Engineering ideas behind silicon computer (such as standardized parts, switches, logic gates, input /output device, arithmetic logic unit, control unit, memory, and busses) have been used by synthetic biologists to build computers with biological parts, with the ultimate goal to control data processing in nature.

This idea based thinking led to three distinct, but inter-related approaches, termed natural computing: computing inspired by nature, computer models of nature, and computing with natural materials [14] (Figure 1).

### Data processing in nature

Focusing on information flow can help us to understand better how cells and organisms work [15]. Data processing can be found in nature all down to the atomic and molecular level. Examples are DNA information storage, and the histone code [16]. Moreover, cells have the potential to compute, both intra cellular (e.g. transcription networks) and during cell to cell communication [17]. Higher order cell systems such as the immune and the endocrine system, the homeostasis system, and the nerve system can be described as computational systems. The most powerful biological computer we know is the human brain [18].

### Computing inspired by nature

General systems theory is an important fundament for computer science [1]. Interesting work has be done, as discussed above, by the Biological Computer Laboratory led by Heinz Foerster [8] [9].

In practical terms, nature inspired to programming paradigms such as cellular automata, artificial neural networks, evolutionary algorithms, evolutionary biology, genetic programming, swarm intelligence, artificial immune systems, membrane computing and amorphous computing [14] [19]. The common aim of all these concepts is solving complex problems.

### Computer models of nature

The aim of the simulation and emulation of nature in computers is to test biological theories, and provide models that can be used to facilitate biological discovery. Moreover, these models can potentially be used for computer aided design of artificial biological systems.

Systems biology provides theoretical tools to model complex interactions in biological systems [12]. Design principles of biological circuits have been translated into mathematical models. These design models find their practical application in synthetic biology in general, and cellular computer especially. The different areas of natural computing clearly influence each other.

A breakthrough in the modeling and synthesis of natural patterns and structures was the recognition that nature is fractal [14]. A fractal is a group of shapes that describes irregular and fragmented patterns in nature, different from Euclidean geometric forms [20].

Other mathematical systems, as cellular automata, are both inspired by nature and can be used to modulate nature *in silico*, as some biological processes occur, or can be simulated, by them such as shell growth and patterns, neurons and fibroblast interaction [21] [22].

Another computational model of nature is the Lindenmayer-system (or L-system), which is used to model the growth process of plant development [23]. A major step towards the creation of artificial life was recently achieved by Karr et al [24]. This group reports a whole-cell computational model of the life cycle of the human pathogen Mycoplasma genitalium that includes all of its molecular components and their interactions. This model provides new insight into the *in vivo* rates of protein-DNA association and an inverse relationship between the durations of DNA replication initiation and replication. Moreover, model predictions led to experiments which identified previously undetected kinetic parameters and biological functions.

### Computing with natural materials

Engineering ideas behind silicon computers can be applied to engineering with natural materials in order to gain control over biological systems. This concept started to emerge in the 1960s when Sugita published ground breaking theoretical work where he performed a functional analysis of chemical systems *in vivo* using a logical circuit equivalent [25] [26]. He discussed the idea of a molecular automaton, the molecular biological interpretation of the self-reproducing automata theory, and the chemico-physical interpretation of information in biological systems [27] [28]. Sugita made analogies between an enzymatic cascade and logic, values and concentrations, and interactions and circuit wires.

The emerging field of synthetic biology has contributed with novel engineering concepts for biological systems [29] [30]. The development of standardized biological parts has been a major task in synthetic biology, which led among other things to the open MIT Registry of Standard Biological Parts, and the BIOFAB DNA tool kit [30] [31] [32]. Another engineering principle, abstraction hierarchy, deals with the question of how standardized parts build a complex system. Systems (systemics) are another important engineering paradigm dealing with complexity [9] [33]. A system is a set of interacting or independent components forming an integrated whole. Common characteristics of a system are: components, behaviors and interconnectivity. Systems have a structure defined by components. Systems behavior involves input, processing and output of data. Behavior can be described with terms such as self-organizing, dynamic, static, chaotic, strange attractor, adaptive. Systems have interconnectivity. This means that the parts of the system have functional as well as structural relationships between each other. This kind of thinking represents a move form molecular to modular biology [34]. The challenge is to define the hierarchical abstraction for such a modular system for biocomputers, and finally actually build such a system.

A breakthrough paper was published in 1994 by Leonard Adleman [35]. For the first time a biocomputer, based on DNA, was built. This system was able to solve a complex, combinatorial mathematical problem, the directed Hamiltonian path problem. This problem is in principle similar to the following: Imagine you wish to visit 7 cities connected by a set of roads. How can you do this by stopping in each city only once? The solution of this problem, a directed graph was encoded in molecules of DNA. Standard protocols and enzymes were used to perform the “operations” of the computation. Other papers using DNA computing for solving mathematical problems followed [36]. Adelman's paper basically kick started the field of biological computers (reviewed in [17] [18] [37] [38] [39]).

## Biological parts as system components for biocomputers

A system consists of defined components. In order to build a biocomputer system, we need to identify these components and standardize them. Although important work is done in synthetic biology in respect to part standardization in general, for biocomputer parts this work is so far rudimentary. Thus, we will try in the following to identify and classify them. Such standardized biological parts suitable for computing can be found all along the line of the central dogma of biology: DNA, RNA, protein, and cells (Table 1 to 4).

**Table 1:** DNA based parts and their application in biocomputing. Representative references are provided.

Part	Circuit	Switch	I/O	Arithmetic Logic	Control Unit	Memory	Buss
nucleotides (order)						41 - 45	
DNA (recombination)				46, 47		42, 45	
DNA (hybridization)				95, 133, 143, 146, 147, 148			
DNA (self-assembly, tiling)				50	18		
gene regulatory circuit / network	67 - 72	116		73, 112, 123 - 130		116	
transcription factors				75	74		
combinatorial promoters				77			
aptamers				131, 132	157		
deoxyribozyme (DNAzymes)				80, 127, 128, 134			
I-switch		117					
transcriptor				135			

### DNA

The natural function of DNA is to store hereditary information and regulate the expression of this information [40]. Following the Adelman paper a wide range of DNA properties suitable for computing were explored. DNA may serve either as a principal structural component, or as a mediator that arranges tethered ligands or particles [40].

Structural properties of the DNA as the order of nucleotides, recombinational behaviors, self-assembly due to Watson-Crick base paring and storage of free energy have been used for different aspects of computational systems (see Table 1).

Nucleotide sequence: The order of nucleotides within a DNA molecule can be used to store information [41] [42] [43] [44] [45].

DNA recombination: Recombinational DNA behavior, allowed by specified classes of enzymatic activities, has been described in terms of the formal language theory, a branch of theoretical computer science [46]. The associated language consists of strings of symbols that represent the primary structures of the DNA molecules that may potentially arise from the original set of DNA molecules under the given enzymatic activities. Moreover, DNA recombination has been used to [47] solve a mathematical problem: sorting a stack of distinct objects (genetic elements) into proper order and orientation (site-specific DNA recombination) using the minimum number of manipulations [47].

Self-assembly: DNA can self-assemble through Watson-Crick base pairing to produce an arrangement of tiles (shapes) that covers the plane [48]. Computation by tiling is universal, because tiles and matching rules can be designed so that the tilings formed, correspond to a simulation of that device [49]. Thus, macroscopic self-assembly of different DNA-based tiles can be used to perform DNA-based computation. This was e.g. demonstrated by building a one-dimensional algorithmic self-assembly of DNA triple-crossover molecules that can be used to execute four steps of a logical XOR (if either input 1 or input 2 is true (1), so output true; if all input are false (0) or all input are true, so output false) operation on a string of binary bits [50]. Chemically, the value of a tile, 0 or 1, is denoted by the presence of a restriction site (eg Pvu II represents 0, false and EcoR V represents 1, true). Each molecular tile contains a reporter strand in order to extract the answer after self-assembly occurred. The answer produces a barcode display on an analytic gel. This system is static as self-assemble results into prescribed target structures. However it is also possible to engineer transient system dynamics such as in self-assembly pathways. It has been shown that it is possible to program diverse molecular self-assembly and disassembly pathways using a ‘reaction graph’ abstraction to specify complementarity relationships between modular domains in a versatile DNA hairpin motif [51]. Programming of functions such as a catalytic circuit, nucleated dendritic growth, and autonomous locomotion were achieved with this approach. Moreover, even something sophisticated such as barcodes have been engineered from self-assembled DNA [52].

Free energy stored in DNA: The hydrolysis of the DNA backbone and strand hybridization, are spontaneous because they are driven by the potential free energy stored in DNA itself. A molecular computer using these operations may, in principle, be fueled by its DNA input. Thus it is possible to use the potential energy of a DNA input molecule to drive molecular computation [40] [53] [54].

As mentioned, another way DNA may function in biocomputers is as a mediator that arranges tethered ligands or particles [40].

Transcriptional regulatory circuits: A cell senses its environment and calculates the amount of protein it needs for it various functions. This information processing is done by transcription networks. These networks, a major study object of systems biology, often contain recurring network topologies called ‘motifs’ [55]. Composition and engineering concepts for these circuits have been extensively studied [56] [57] [58] [59] [60] [61] [62] [63] [64] [65] [66]. Many interesting functions such as oscillators, frequency multipliers and frequency band-pass filter have been engineered [67] [68] [69] [70] [71] [72]. Transcriptional regulatory circuits can be seen as an analog to electronic circuits. Data input, data processing and data output is an abstraction found in both circuit types. Transcriptional circuits have chemicals as an input. Data processing happens as functional clusters of genes impact each other's expression through inducible transcription factors and cis-regulatory elements. The output is e.g. proteins. Diverse computational functions (see below) have been engineered through changes in circuit connectivity [73].

Transcription factors: Trigger-controlled transcription factors, which independently control gene expression, have been used as part of the processing unit in a programmable single-cell mammalian biocomputer [74]. Artificial Cys(2)-His(2) zinc finger transcription factors specifically bind different DNA sequences and thus provide components for designing of regulatory networks^75^.

Combinatorial promoters: Promoters control the expression of genes in response to one or more transcription factors. Rules for programming gene expression with combinatorial promoters have been identified [76]. This opens the option to engineer a wide range of logic functions. Both Boolean and non-Boolean logic is possible as the concentration of regulators is not necessary binary. As an example, a combinatorial promoter has been engineered, which expresses an effector gene only when the combined activity of two internal input promoters is high [77].

Enzymatic machinery for DNA manipulation: Novel cleavage specificities have been designed by combining adapter oligodeoxynucleotide and enzyme moieties [78]. Moreover, functional higher-order nucleic acid complexes can be built from modular motifs such as aptamers (a DNA molecule that specifically binds a small molecule or biomolecule), aptazymes (a DNA molecule that is comprised of an aptamer domain fused to a catalytic domain) and deoxyribozymes (DNAzymes, a DNA molecule with catalytic properties) [79]. This kind of design results in highly programmable, smart complexes, which enable engineering beyond conventional genetic manipulation. In line with this, a DNA-based computational platform has been constructed that uses a library of deoxyribozymes, and their substrates, for the input-guided dynamic assembly of a universal set of logic gates and a half-adder/half-subtractor system [80].

Dynamic constructs formed by DNA: Furthermore, DNA can be used to engineer dynamic constructs such as molecular switches and oscillating molecular machines (see below).

### RNA

Another promising approach for building biocomputers uses RNA molecules and RNA-based regulation [81]. RNA editing, the modification of RNA sequences, can be viewed as a computational process [37]. Moreover, RNA is involved in regulatory networks, which have been described as normal forms of logic function in the form of: input, logic gate and output [81] [82]. In many RNA based computational systems the inputs are often small RNA molecules or motifs, while the output is mRNA [81] [83] [84]. Different classes of regulatory RNA components for engineering such systems, have been identified e.g. RNA aptamer, ribozymes, riboswitches, orthogonal ribosomes, miRNA and siRNA (Table 2) [85].

**Table 2:** RNA based parts and their application in biocomputing. Representative references are provided.

Part	Circuit	Switch	I/O	Arithmetic Logic	Control Unit	Memory	Buss
RNA library / ribonuclease				86			
aptamer			87, 88	88, 136			
ribozyme			88	88, 138, 139			
riboswitch / riboregulator		79, 92, 93, 119, 120, 137		137			
RNA (hybridization)				97, 98			
amber suppressor tRNA				140, 141			
orthogonal ribosomes				94			
miRNA			84	84, 95 - 97			
siRNA / shRNA		121	98	97, 98, 121			
CRISPR associated Cas9					99		

Binary RNA library and ribonuclease (RNase) H digestion: The Adleman molecular computing approach has been expanded to RNA [86]. Using specific ribonuclease digestion to manipulate strands of a 10-bit binary RNA library, a molecular algorithm was developed and applied to solve a chess problem.

RNA aptamer: A RNA molecule that specifically binds a small molecule or biomolecule has been engineered to function as an input sensor in biological computing devices [87] [88].

Ribozymes: Catalytic RNA, ribozymes, can play an interesting role in biocomputing [89] [90]. In general, ribozyme activity (cleavage) in cis will repress translation, whereas activity (cleavage) in trans may repress or activate translation [85]. The hammerhead ribozyme is a small, naturally occurring ribozyme that site-specifically cleaves RNA [91]. This ribozyme can function as an actuator in a RNA computing device [88]. Input binding is translated to a change in the activity of the actuator, where a “ribozyme- active” state results in self-cleavage of the ribozyme [88]. The RNA device is coupled to the 3′ untranslated region of the target gene, where ribozyme self-cleavage inactivates the transcript and thereby lowers gene expression [88]. Different signal integration schemes act as various logic gates.

Riboswitches: Regulatory RNA elements can act by binding a small molecule, and thus switching gene expression on or off [92] [79] [93]. Ligand binding may repress or activate transcription or translation [85].

Orthogonal ribosomes: Multiple unnatural (orthogonal - O) ribosomes can be used combinatorially, in a single cell, to program Boolean logic functions [94]. O-ribosomes functioned as input, O-mRNAs as logic gate and fluorescence as output.

miRNA: Binding of microRNA (miRNA) represses translation [85]. This makes miRNA suitable to serve as sensory module to DNA-based digital logic circuits [95] [84] [96] [97].

siRNA: Small interfering RNAs (siRNA) are a class of short RNAs that stimulate post- transcriptional gene silencing through the RNA interference (RNAi) pathway in higher eukaryotes [85]. RNAi has been used to construct a synthetic gene network that implements general Boolean logic to make decisions based on endogenous molecular inputs [98] [97]. The state of an endogenous input was encoded by the presence or absence of ‘mediator’ small interfering RNAs (siRNAs).

Programmable DNA/RNA editing: Recently, a new kind of endonuclease has been discovered which can potentially play an interesting role in the design of biocomputers. This endonuclease is the CRISPR (Clustered regularly interspaced short palindromic repeats) associated Cas9 [99]. Cas9 forms a complex with dual-RNAs. The RNA sequence defines a site-specific DNA binding of this complex. This results dsDNA cleavage. This system has a potential for RNA-programmable genome editing. Another RNA-editing platform has been developed by using the bacterial CRISPR pathway [100]. This enables predictable programming of gene expression.

### Proteins

Protein based logic systems have been generated *in vitro* [101]. Furthermore, recent studies have developed strategies for protein synthetic biology *in vivo* [102]. Proteins play both as input and output signals a crucial role in the information processing in the cell. Moreover, logic can also be implemented by the regulation of protein functions governing the production, destruction, localization, and activities of biochemical molecules [102] [103] (Table 3).

**Table 3:** Protein based parts and their application in biocomputing. Representative references are provided.

Part	Circuit	Switch	I/O	Arithmetic Logic	Control Unit	Memory	Buss
enzymes			104 - 107	101, 104, 105, 106, 143			
transactivator / transrepressor				74, 108			
protein (chemically inducible dimerization)				109, 110			
DNA polymerase					152, 153		
restriction nuclease FokI, T4 DNA ligase					154		
recombinase / integrase / excisionase						42, 45	
T7 RNA polymerase				142			
zink finger transcription factor				75			

Enzymes: We have already discussed some roles of enzymes in biocomputing e.g. in DNA manipulation. Another interesting concept for engineering an *in vitro* protein-based logic system is based on input and output of enzymatic reactions [104] [105] [106] [107]. Different enzymes were used alone or coupled to construct different logic gates. The added substrates for the respective enzymes, act as the gate inputs, while products of the enzymatic reaction are the output signals that follow the operation of the gates.

Transactivator/transrepressor: Transcription control in mammalian cells can be enabled by logic gates [74]. It has been shown, that chimeric promoters containing operators specific for up to three different transactivators/transrepressor enable NOT and AND-type regulation profiles with three molecular intervention levels [108].

Chemically inducible dimerization (CID): In CID systems, a small molecule induces the dimerization of two different proteins, producing a ternary complex [101]. Such a system has been used to engineer a transcriptional logic device [109]. A major drawback of many engineered logic circuits is that they require minutes to hours to execute their logic functions due to the long processing time of the transcription and translation machinery [101]. Non genetic circuit devices based on CID might be able to overcome this obstacle. Such a rapid logic device has been built by Miyamoto et al [110]. Boolean logic gates were synthesized by using two chemical inputs. These gates produced output signals such as fluorescence and membrane ruffling on a timescale of seconds.

### Cell to cell communication

Inter cellular signaling can be used to build logic into biological systems. An interesting aspect lies in compartmentalization of the circuit where all basic logic gates are implemented in independent single cells that can then be cultured together to perform complex logic functions [111]. Such systems are possible in a wide variety of settings. Examples are cell to cell communication in bacteria by quorum sensing and artificial neural networks (Table 4).

**Table 4:** Cell to cell communication based parts and their application in biocomputing. Representative references are provided.

Part	Circuit	Switch	I/O	Arithmetic Logic	Control Unit	Memory	Buss
quorum sensing							112
biological neural networks				113, 114			

Quorum sensing: Quorum sensing is a system used by many species of bacteria to coordinate gene expression according to their population density. A simple genetic circuit has been combined with quorum sensing to produce more complex computations in space [112]. Biological neural networks: Biological neural networks are composed of circuits of biological neurons. This has not to be confused with the artificial neural networks we described above, which are programming constructs that mimic the properties of biological neurons. Biological neurons have been used to engineer logic gates [113] [114].

## A biological microprocessor

**Figure 2:**
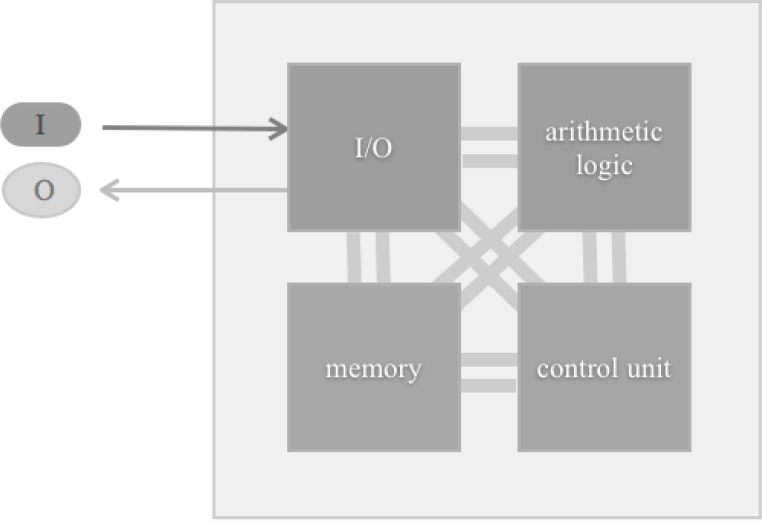
Four units of a general purpose computer: Input and output device (I/O; I = input signal; O = output signal), the arithmetic logic unit, control unit, memory. Busses (groups of wires) connect these units.

We now move from system components to the complete biocomputer system, and define the general purpose silicon computer system as a template for biocomputers. Such a template consists of four units: the input and output device (I/O), the arithmetic logic unit, the control unit and the memory (Figure 2) [115].

The first three units collectively build the central processing unit (CPU), typically constructed on a single integrated circuit called a microprocessor. The control unit coordinates the various system components. It decodes the program instructions, and transforms them into control signals, which activate other system parts. This finally results in a change of the system state. Historically the control unit was defined as a distinct part, whereas in modern design this unit is an internal part of the CPU. Busses (often made of groups of wires) interconnect these units. Each unit contains a huge number of small electrical circuits. Switches can turn these circuits on (1) or off (0). A logic gate can perform a logic operation on one or more of such logic inputs and produce a single logic output. Thus, basic elements of any biocomputer unit are switches and logic gates.

### Switches

As discussed the basic function of a switch is to produce an on or off state. Such switches have been engineered based on transcription regulation, artificial DNA, or RNA.

The DNA based type can either be based on a gene regulatory circuit or on DNA molecule properties. The toggle switch, a synthetic, bistable gene-regulatory network in Escherichia coli, belongs to the first category [116]. This toggle switch is a quite famous one, published in a landmark paper, which helped to kickstart synthetic biology. The toggle is constructed from two repressible promoters, such as that repressor 1 inhibits transcription from promoter 1 and is induced by inducer 1, whereas repressor 2 inhibits transcription from promoter 2 and is induced by inducer 2. The switch can take two stable states, if the inducers are absent: one in which promoter 1 transcribes repressor 2, and one in which promoter 2 transcribe repressor 1. The switch is flipped between these stable states by transient chemical or thermal induction of the currently active repressor. All together, the toggle switch forms an addressable cellular memory unit.

Another type of switch, called I-switch, an artificial DNA nano-device, that has cytosine-rich regions, which act as a sensor for chemical input in the form of protons and functions as a pH sensor based on fluorescence resonance energy transfer (FRET) inside living cells. [117]. The I-switch consists of three oligonucleotides, where two with single stranded overhangs are hybridized onto the adjacent third. At acid conditions these overhangs are protonated, leading to a closed conformation with high FRET. This switch was used to map spatial and temporal pH changes during endosome maturation. These experiments demonstrate the potential of DNA scaffolds responsive to triggers in living cells. These principles might be applied to switches in DNA or RNA scaffolds which assemble proteins [118].

We have already discussed one kind of RNA based switche, the riboswitche, above. Another approach is switches based on an engineered riboregulator, which enable post-transcriptional control of gene expression [119]. This riboregulator is constructed such that a small sequence, complementary to the ribosome binding site (RBS), is inserted downstream from a promoter and upstream from the RBS. After transcription a stem loop is formed at the 5‘ end of the mRNA, which blocks ribosome docking and translation. This mRNA can be targeted by another non-coding RNA and undergo a linear-loop interaction, that expose the obstructed RBS and thus activates expression. Interestingly, this kind of artificial riboregulator have been used to build a genetic switchboard that independently controls the expression of multiple genes in parallel [120].

**Figure 3:**
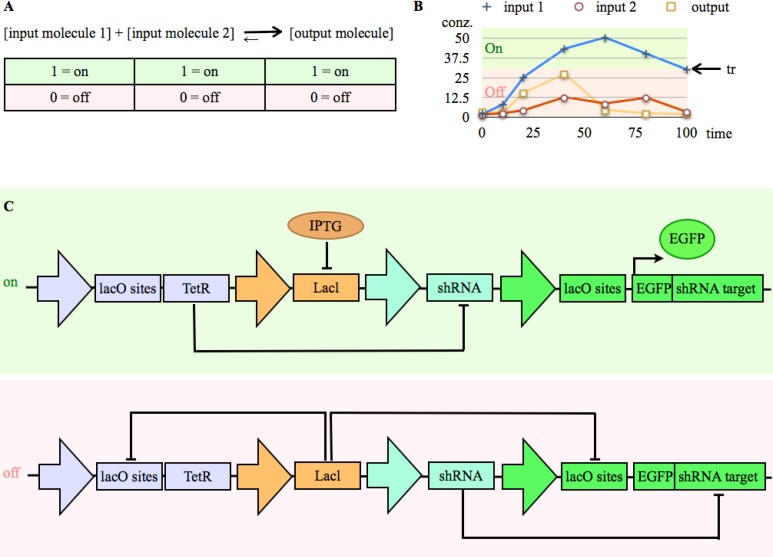
Input/Output (I/O) device: **A)** In a “digital” biological I/O device input molecules induce due to a set of non-steady state chemical reactions (engineered coherent with a logic scheme) an output molecule. All molecules have a defined concentration translated into Boolean logic; alternative on (1) or off (0). **B)** In order to do so, normalized molecule concentrations (conz.), which change over time, are defined as off (0), if they are under a certain threshold (tr), and, if they are above, as on (1) **C)** A switch, which produce an on (induced) or off (not induced) state: The figure gives an example of a switch in a synthetic gene network (adapted from [121]). Off (no detectable EGFP expression): LAcl repressor proteins, which are constitutively expressed, bind to two introns with lac operator (lacO) sites, inducing transcriptional repression of EGFP and TetR respectively. Repression of TetR allows transcription of shRNA, which can subsequently bind to its target sequence, and repress it's shRNA target. On (EGFP expression induced): isopropyl-b-thiogalactopyrano (IPTG) binds to Lacl proteins. As a consequence, the repressor proteins are inactive, as they change their conformation. Thus, TetR, which represses shRNA, and EGFP get transcribed.

As mentioned above, it is possible to engineer Boolean logic based on RNAi. A tunable switch has been built based on a synthetic gene network that couples repressor proteins with a design involving shRNA (Figure 3C) [121].

Although protein based switches, that do not comprise transcription factors, are not uncommon in nature, they have been so far not a major focus [18].

### Logic gates

**Figure 4:**
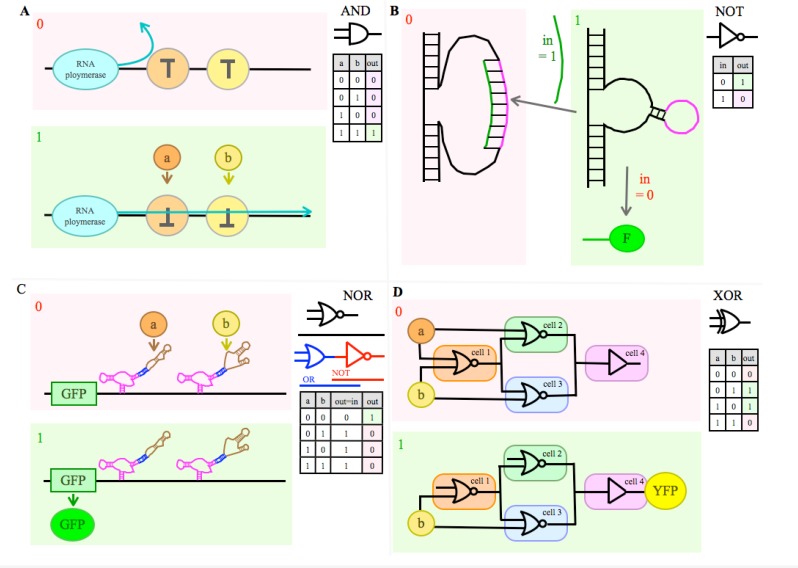
Arithmetic logic unit: Shown are four basic Boolean logic gates (AND, NOT, NOR, and XOR), their symbols and respective truth Table 1 means that the input (a, b) is sensed or the output (out) is released, whereas 0 means not. In the examples system output = 0 is highlighted as pink, output = 1 as green. **A)** An AND gate can be based on the transcriptor (T), an asymmetric transcription terminator, which can block RNA polymerase flows one directional. If both terminators are flipped, induced by their respective input signal (a and b), RNA polymerase flows unhindered (full length RNA output). **B)** Deoxyribozyme based NOT gate: The deoxyribozyme (DNA based catalyst) is in an active form, if no input (in) is present (in = 0). Cleavage activity results in this case in a fluorescent oligonucleotid (F) as output. An oligonucleotide input (in) (in = 1) leads to hybridization of the input strand (green) with the closed loop strand, which is marked purple. This results in an inactive, open loop and the absences of a fluorescent product. **C)** An RNA aptamer based NOR gate: NOR is an OR gate followed by a NOT gate. Two subsequent RNA devices consist, is this case, each of three functional components: a sensor, made of an RNA aptamer (brown), an actuator component, made of a hammerhead ribozyme (purple), and a cobbling sequence between these parts, the transmitter (blue). Translation of the gene of interest (here GFP), encoded upstream of the device, is only possible in the case of the absence of both inputs (a and b). **D)** An inter cellular network based XOR gate: The system is built from four Escherichia coli colonies, whereas each colony consists of a strain engineered to contain a single gate. Three cell colonies (cell 1, 2, 3) containing NOR gates and a fourth (cell 4) a BUFFER gate (two subsequent NOT gates; if in = 0, so out = 0; or if in = 1, so out = 1). The cell colonies communicate through quorum sensing, which represent the “wires” of the system. If both inputs (a, b) are present, or if a and b are absent, the system has no output. If either a or b is present, yellow fluorescent protein (YFP) is expressed.

A logic gate is an elementary building block of a digital circuit. These gates can have one or two inputs, but only one output. Inputs and output are of Boolean nature, thus they can be either true (1) or false (0). Different logic operators can be applied on the input. Basic types of logic gates are: AND, OR, NOT (inverter), XOR, NAND, NOR, and XNOR [101] [18]. These operators are the basis for different truth tables (Figure 4). We get a true output from the gate for the following case: AND - both inputs are true; OR - either or both inputs are true; NOT - (has only one input) if the input is false; XOR (either/or) - either input 1 or input 2 is true; NAND - (is an AND gate followed by a NOT gate) both inputs are false, or one is true; NOR (OR followed by NOT) - both inputs false; and XNOR (XOR followed by NOT) - both inputs are true or both are false. All other cases give a false output respectively. Over a period of about two decades DNA, RNA and protein based logic gates have been engineered and classified [122] [101]. A wide range of core machinery and inducers has been developed.

DNA based logic gates: One strategy for engineering a logic gate *in vivo* is to build a core machinery, based on gene expression regulation [123] [124] [125] [126] [127] [128] [129]. One such system had two inputs such as beta-D-thiogalactopyranoside and anhydrotetracycline (aTC) and a fluorescent protein as output [73]. In order to build such a logic system, a network plasmid was generated composed of a set of three transcription factor encoding genes (LacI, TetR, and lambda cI) and their corresponding promoters. The binding state of LacI and TetR can be changed with the input molecules. Moreover, the system consists of five additional promoters which can be regulated by the three transcription factors. Two of the promoters are repressed by LacI, one is repressed by TetR, and the remaining two are respectively positively or negatively regulated by lambda cI. Altogether, this results in 125 possible networks. Various GFP expressing systems can be formed using a combination of various promoters, input molecules and host strains e.g. E.coli. In such manner functional networks were formed with logic operations such as NOR, NOT, and NAND.

Another option to build a core machinery both *in vivo* and *in vitro* is by using DNA aptamers [130] [131]. Yoshida et al built an AND gate by fusing an adenosine-binding DNA aptamer and a thrombin-binding DNA aptamer [130]. Each aptamer binds to partially complementary fluorescence quencher-modified nucleotides, QDNA1 and 2 respectively. When the two inputs adenosine and thrombin are bound both QDNAs are released from the aptamers leading to increased fluorescence intensity. Other input combinations (0 + 0, 0 +1, and 1 +0), lead to the presence of zero or one QDNA and a weaker fluorescence. Similar, an OR gate can be created, if the positions of the fluorophore and QDNA are modified. Another study built an aptamer based nanorobot, which has an open and closed conformation [131]. DNA aptamer–based lock mechanism opens in response to binding of antigen keys. This lock functions as an AND gate, where the aptamer-antigen activation state serves as input, and the nanorobot conformation as output.

Hybridization can serve as another *in vitro* option for engineering a core machinery feasible for functioning in a logic based network [95] [132]. A two input logic gate of type AND, OR or NOT were constructed by using a branch-migration scheme with a mechanism built on strand recognition and strand replacement. Single stranded nucleic acids are input and output of such a scheme. The gate function is created by sequential base pairing triggered by toehold-toehold binding between single strands and subsequent breaks.

Moreover, *in vitro* deoxyribozyme-based (DNAzymes) logic gates have been engineered [127] [128] [133]. In order to engineer an AND gate, two different oligonucleotide inputs were hybridized with corresponding controlling elements [127]. This led to the cleavage of the substrate in the presence of both inputs and subsequent conformational change of controlling elements. A NOT and XOR gate was constructed in a similar fashion (Figure 4B).

Recently, a novel *in vivo* system, called transcriptor, has been used to build permanent amplifying AND, NAND, OR, XOR, NOR, and XNOR gates to control transcription rates (Figure 4A) [134].

RNA based logic gates: The other major class of logic gates is RNA-based. The core machine can be based on RNA aptamer, a riboswitch, ribozymes, hybridization, amber suppressor tRNA, or an orthogonal ribosome [101].

Culler et al demonstrated *in vivo* that it is possible to engineer an AND gate based on a β-catenin binding RNA aptamer [135]. This aptamer was inserted into the intron position, between a protein-coding exon and an alternatively spliced exon (Ex) containing a stop codon, followed by another intron, the next protein-coding exon and the herpes simplex virus- thymidine kinase (HSV-TK) gene whose product, in turn, is an activator of ganciclovir (GCV). Binding of β-catenin with the RNA aptamer led to mature mRNA which lacked Ex. This led to the expression of HSV-TK. If the alternatively spliced exon was not excluded from the mature mRNA, an early translation termination occurred. This resulted in the synthesis of a nonfunctional peptide. For the induction of apoptosis as output, both expression of HSV-TK and the presence of GCV are required. Another study from the same lab demonstrated the building of AND, NOR, NAND, or OR gates based on RNA aptamer as a core machine (Figure 4C) [88].

We already discussed a simple riboswitch as a structure suitable to build logic. Sudarsan et al. reported a tandem riboswitch core machinery *in vivo* that facilitate more sophisticated control [136]. They discovered in the 5’ untranslated region of Bacillus clausii metE RNA two naturally occurring riboswitches. Both riboswitches bind independently to two different metabolites, one to S-adenosylmethionine (SAM) and the other to coenzyme B12 (AdoCbl). This binding induced the transcription termination of gene of interest through cis-acting corresponding riboswitches. Only in the absence of both inputs (not SAM and not AdoCbl) we get the full length transcript as output. All together this system functions as a two-input Boolean NOR logic gate.

Logic gates have been engineered with ribozymes as core machinery both *in vivo* and *in vitro* [137] [138]. An AND is built when simultaneous hybridization of two oligonucleotide inputs with the ribozyme lead to its activation [137]. Chen et al engineered a YES gate (if input 0 so output 0; if input 1 so output 1) in a system based on a ribozyme, which was inserted into the 3′-UTR of a target transgene [138]. The ribozyme was inactivated in the presence of theophylline, allowing the target transgene to be expressed.

Alternatively a logic gate can be based *in vivo* on hybridization, with siRNA or miRNA as input [98] [97]. An AND like logic function has been built by using two groups of miRNAs as input and the hBAX protein as output [97]. The miRNAs act as a repressor of activators and repressors in the gate.

Amber suppressor tRNA can be used *in vivo* as the core machinery for a logic gate [139] [140]. This kind of tRNA identifies the “amber” stop codon (UAG), inserts an amino acid, and do not terminate translation. Anderson et al. utilized an amber suppressor tRNA (SupD) to engineer a two input AND gate [140]. One input is a salicylate responsive promoter, which is linked to the transcription of the amber suppressor tRNA supD. The other input is a arabinose responsive promoter, that regulates the transcription of T7 RNA polymerase. T7 has been mutated to contain two amber stop codons and thus requiring SupD expression for a fully functional T7, which is connected to the expression of green fluorescent protein as an output.

Furthermore, an AND gate has been engineered *in vivo* using an orthogonal (unnatural) ribosome / mRNA pair [94]. The inputs in this system are two orthogonal rRNAs, which limit the translation of two respective mRNAs. These mRNAs encode two fragments of beta-galactosidase, which's activity is the output of the system.

Protein based logic gates: The third class are protein based logic gates, where a transactivator, an enzyme, chemically inducible dimerization (CID), a T7 RNA polymerase or a zink finger transcription factor can act as core machinery in a logic gate [101] [141].

Transactivator: Various logical gates have been engineered *in vivo* based on chemically inducible transactivator-based gene circuits [108] [74]. This conception was used by Ausländer et al. to construct several logic gates and combination of them, such as NOT, AND, NAND and N-IMPLY (if a = 0 and b = 1, so output = 1; else output = 0) [74]. Such a N-IMPLY gate was engineered by combining an erythromycin-dependent transactivator and an apple metabolite phloretin-dependent transactivator. The output, fluorescent d2EYFP, was only visible by fluorescent microscopy or FACS analysis in the presence of erythromycin and absence phloretin.

Enzyme based logic gates, such as XOR, N-IMPLY, AND, OR, NOR, NOT, and YES (one input; if input =1, output =1; else output = 0), have been constructed for *in vitro* systems with a wide variety of inputs, such as glucose, H2O2, NADH, acetaldehyde, starch, phosphate, NAD+, acetylcholine, butyrylcholine, O2 [101] [104] [105] [106] [142]. Baron et al. constructed eg a two input AND gate [104]. Both H2O2 and glucose are in this case necessary input in order to activate the catalytic chain with gluconic acid as output.

Another option for building Boolean logic *in vivo* is based on a CID system [109] [110]. Bronson et al. utilized a CIT system to engineer a two input AND gate [109]. Dexamethasone– methotrexate input induced the dimerization of an activation domain, B42-glucocorticoid receptor chimera (B42-GR), and a DNA-binding domain, LexA-dihydrofolate reductase chimera (LexA-DHFR). Both B42-GR and LexA-DHFR expression is placed under the control of the GAL1 promoter. Thus galactose is required as second input in the system in order to form the ternary complex. This complex induces, as output of the system, acts as a transcriptional activator and stimulates the transcription of the output, a lacZ reporter gene.

Recently Shis et al. published another interesting option to build an AND gate [141]. A functional T7 RNA polymerase can be built from two fragments, whereas the larger T7 RNA polymerase fragment is encoded by a gene that responds to arabinose and the smaller fragment by a gene that responds to lactose. T7 RNA polymerase will be functional active in the presence of both inputs, arabinose and lactose.

OR, NOR, AND and NAND logic has been based on artificial Cys2– His2 zink finger (ZF) transcription factors as computing elements [75]. Input signals led to expression of corresponding ZF-based transcription factors, which acted on response promoters. An OR gate was constructed, which contained target sites for two different ZF activators [75]. BCR_ABL-1:GCN4 and erbB2:Jun activators were used as ZF-1 and ZF-2, respectively. AmCyan fluorescent protein output, measured by flow cytometry, was observed, when either or both inputs were present.

Cell to cell communication based logic gates: Logic systems built on gene expression regulation can be expanded to multicellular engineered networks [112] [143]. Different logic gates were carried in one study by different strains of E.coli, which communicate by quorum sensing (see above) (Figure 4D) [112]. Input was aTC or arabinose. Colonies containing different gates were wired together via quorum molecules. Different combinations of colonies containing specific simple logic gates resulted in the construction of 16 two-input Boolean logic gates. Different combinations of 2 input molecules such as NaCL, galactose, 17 beta-estradiol, doxycycline, galactose, or glucose were used in another study which builds a multicellular network based on gene expression regulation [143].

Finally, one might ask how many gates can be interconnected with the present technology in a circuit. A study by Privman et al. tried to determine this maximum number under optimal noise reduced conditions [144]. They concluded that under such conditions, logic gates can be concatenated for up to order 10 processing steps. Beyond that, it will be necessary to engineer novel systems for avoiding noise buildup.

### Input and output (I/O) device

A biomolecular I/O device is basically an engineered set of chemical reactions with input and output molecules with distinct concentrations, formalized as e.g for the case of a two input device: [input molecule 1] + [input molecule 2] <-**>** [output molecule] (Figure 3A).

In order to act in a digital manner, the concentrations need to be defined as distinguishable high or low, which can be translated to Boolean logic (low as 0 or of, and high as 1 or on) (Figure 3B). As we already discussed above, a variety of interesting devices have been constructed (Figure 3C) [79] [92] [93] [116] [117] [119] [120] [121] [137]. However, reaction kinetics and dynamics are often difficult to predict as values in a living cell are often continuous, can variate to a certain degree, are away from a steady state, and can be difficult to quantitate [18]. Thus, to facilitate Boolean logic, thresholds of inputs and outputs must be well defined, which can be difficult to achieve in biological systems [18]. Depending on the kind of system this can this be concentrations, localization of biomolecules or enzyme activity [101]. A linear system can contribute to minimize retroactive effects; as such a system allows applying well defined control theory. Oishi et al tried to address these kinds of problems and tried to identify design principles for an ideal linear I/O system [145]. Their implementation of such an I/O systems was based on idealized chemical reactions, and on enzyme-free, entropy-driven DNA reactions.

### Arithmetic logic unit

The arithmetic logic unit performs two classes of operations: arithmetic and logic. Both have been engineered in biological systems.

Biological computers have shown t no be able to execute simple arithmetic such as addition and subtraction, as Ausländer et al have demonstrated by a combinatorial assembly of chemically inducible transactivator-based logic gates [74]. Moreover, it has been shown that more complex arithmetic is achievable. A combination of several DNA hybridization based logic gates make it e.g. possible to calculate the integer part of a square root of a four-bit binary number [132] [146].

Logic can be built, as discussed by means of logic gates (Figure 4).

The ability of biocomputers to solve logic problems beyond the Hamiltonian path problem have been demonstrated by the implementation of several logic requiring games [35] [147] [133] [47] [86]. A molecular automaton was engineered, which was able to play a game which covers all possible responses to two consecutive sets of four inputs [147]. Moreover, a deoxyribozyme-based automation is able to play a complete game of tic-tac-toe [133]. A device based on DNA recombination was able to solve a sorting problem, where a stack of distinct objects needed to be placed into a certain order and orientation using a minimal number of manipulations [47]. A molecular algorithm based on ribonuclease digestion to manipulate strands of a 10-bit binary RNA library has been used to address the so called “Knight problem” which asks what configurations of knights in a chess game can one place on a 3 x 3 chess board such that no knight is attacking any other knight on the board [86]. Moreover, DNA hybridization based logic has been used to implement simple logic programs [148]. This logic system consisted of molecular representations of facts such as Man(Socrates) and rules such as Mortal(X) <-- Man(X) (Every Man is Mortal). The system was able to answer molecular queries such as Mortal(Socrates)? (Is Socrates Mortal?) and Mortal(X)? (Who is Mortal?).

### Control unit / Central processing unit

**Figure 5:**
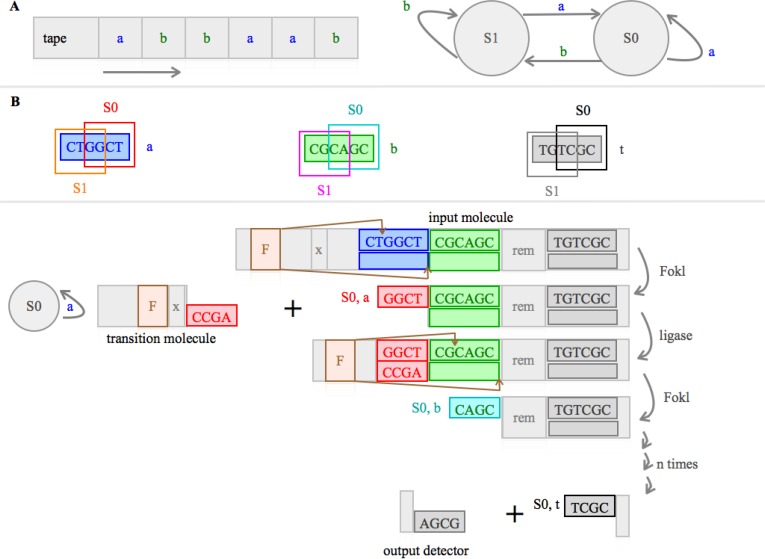
Control unit; central processing unit: **A)** A final state machine, as shown here, is a theoretical model which can help to understand what is going on in the central processing unit. Simplified: Symbols a and b are written on a tape, which is read by the machine letter by letter from left to right. In this example the tape ends with the final letter b. Each letter provides the instruction to the machine into which state (S1, or S0) it should move; here a means move to state state 0 (S0) and b codes the instruction move to state 1 (S1). The final state of the machine in this example is thus S1. **B)** Molecular implementation of a final state machine. The upper part of the figure contains the definitions for this machine: The symbols a, b, and t (terminator) are implemented as a sequence of six specific nucleotides. The state (S1 or S0) of the machine is defined by a 5’ overhang (generated during the computing process, see below) consisting of 4 specific nucleotides (inside the frames). The terminator defines the final symbol read. The machine consists of an input molecule, a transition molecule, an output detector and two enzymes Fokl and ligase. The input molecule consist of a Fokl recognition site (F), a spacer x (a certain defined number of nucleotides), a nucleotide sequence defining a and b, a sequence with the remaining symbols (rem = n numbers of a and b in a defined order) and the terminator sequence (t). Fokl is a restriction endonuclease which can bind to F. It cleaves the DNA (without further sequence specificity) on the sense strand 9 nucleotides downstream and the anti-sense strand 13 nucleotides upstream of the nearest nucleotide of the recognition site. Thus the space x defines where Fokl is cutting. The cleavage of the input molecule results in the first intermediate state (S0), an 5’ overhang, reading a. Ligase ligate this product with the transition molecule. This transition molecule determinates the transition between the states, in this example: if a is read, move from S0 to S0. Other transition molecules can be generated defining all the other possible transition rules. These molecules are designed such that the 4 bases long 3’ overhang reads the symbol, the spacer x defines the cutting point of Fokl 1 and the state the machine will transit to (here S0). The input molecule and the transition molecule get ligated. A new digestion with Fokl leaves an 5’ overhang representing S0 and a reading b. This cycle continues until all remaining symbols (rem) are read and state transitions are executed. The last digestion leaves a 5’ overhang with a terminator sequence defining the final state, in general either S0 or S1 (in this example S0). The molecule in its final state, gets ligated to an output detector, engineered to recognize either state 0 or 1. This forms an output-reporting molecule, which can be detected by gel electrophoresis.

A state machine is a theoretic mathematical model, which helps to understand what is going on in the central processing unit of a computer, and which can be experimentally implemented (Figure 5A) [18] [149] [150].

State is defined as all the stored information, at a given point in time, to which the circuit or program has access. The output of a circuit or program is determined by its input and states. The simplest form of such a state machine is called finite state machine (or finite state automata). In simple terms, this machine contains a tape with symbols a and b. The tape can move in one direction and the machine can read the symbol on the tape. The machine changes its state due to the letter it reads. A string transducer is a state machine that also can write symbols and a Turing machine can in addition move from left to right [151].

State machines have been engineered with biomolecules [152] [153] [154] [40] [155] [156] [157] [158]. Hagiya et al. built in 1997 the first state to state transition system by guiding DNA polymerase based DNA extension by a template strand with a transition rule sequence [152] [153]. The present state is encoded by the 3’-end sequence of a single-stranded DNA molecule. The template strand (rule) enclosed a binding site for the 3-end of the DNA molecule and the extension template. State transition occurred, if the current state is annealed onto an appropriate portion of DNA encoding the transition rules and the next state was copied to the 3’-end by extension with polymerase. The extension template represents the new state.

The first experimental implementation of a finite state machine, comprising DNA and DNA-manipulating enzymes, was published by Benenson et al. in 2001 (Figure 5B) [154] [18]. Similar to the concept developed by Benenson et al. several finite state machine were later developed in which the transitions were executed by autonomous biochemical steps based on DNA sticky end recognition, ligation and digestion [40] [156]. This system was expanded by Adar et al. to allow stochastic computing. The core of this form of computing is the choice between alternative computing paths (biochemical pathways), each with a prescribed probability, which were programmed by the relative molar concentrations of the software molecules coding for the alternatives [155]. Another finite state machine based on DNA aptamer generated different configurations (outputs) in response to a set of two different groups’ chemical inputs [157]. Moreover, by using molecular finite state machines simultaneously with fluorochrome labeled DNA it was possible to distinguish between two distinct images encrypted onto a DNA chip [158].

### Memory

DNA's biological role is to encode huge amounts of data, theoretically up to two bits per nucleotide or 455 exabytes per gram of ssDNA [43]. It has been recently shown, that it is possible to encode arbitrary digital information in DNA, e.g. an html-coded draft of a book that included 53,426 words, 11 JPG images and 1 JavaScript program into a 5.27 megabit bitstream [43]. The oligonucleotides library was engineered by utilizing next-generation DNA synthesis techniques. In order to read the encoded book, the library was amplified by PCR and subsequent sequenced. A similar study encoded computer files totalling 739 kilobytes into a DNA code [44].

Another important feature of DNA is the relatively permanence of the storage. Even after the cells die, one might be able to recover information from the DNA. These storage abilities make DNA suitable as core machinery for engineered memory devices.

**Figure 6:**
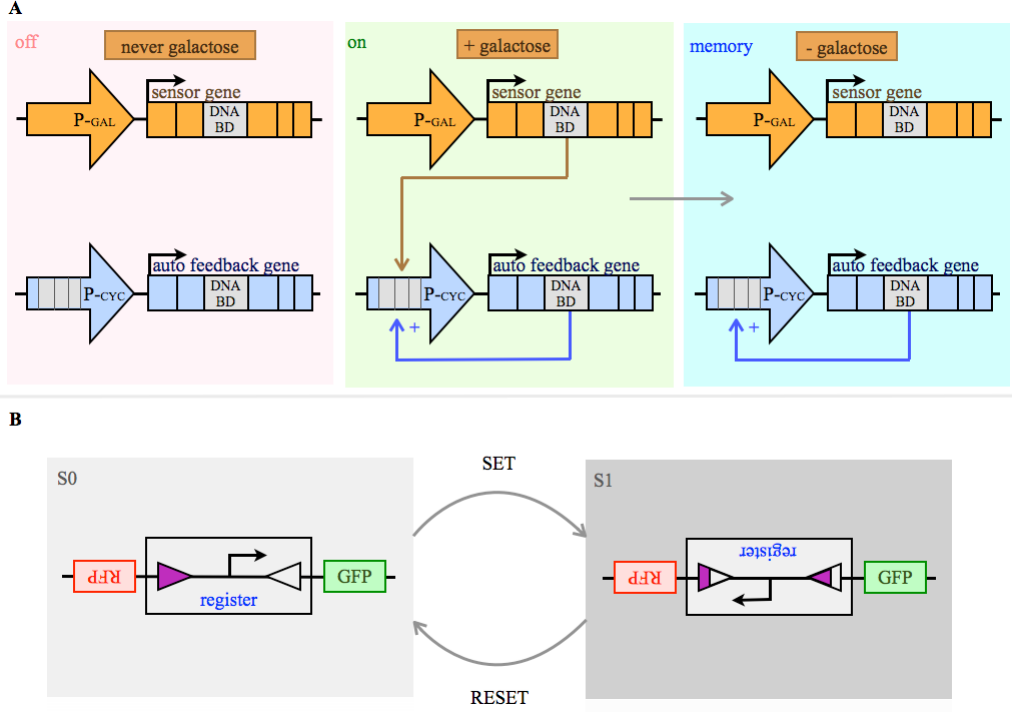
Memory: **A)** Shown is a simplified diagram of a modular memory device, which is a transcriptionally controlled network composed of two transcription factor encoding genes, a sensor gene and a positive (+) auto feedback gene (P-GAL = GAL 1/10 promoter, P-CYC = CYC 1 promoter, DNA BD = sequence encoding a DNA binding domain of the respective transcription factor). The network can be in three states, off, on and memory. The system is in of state, if it has never been exposed to a signal (here galactose). It is on, if galactose is present. In this case the signal induces the synthesis of a transcription factor, the sensor. This triggers the expression of another transcription factor able to bind to its own promoter. The system is in memory state, if the signal is removed. The auto feedback activator is able to initiate its own expression even if the inducing signal is lacking, which means that the system has stored information. **B)** A rewritable recombinase addressable data module, able to store data within a DNA sequence (simplified adaption from [42]): Serine integrase and excisionase are used to invert and restore specific DNA sequences. The system has two potential inputs; a set and a reset transcription signal. This set signal drives expression of integrase which inverts a DNA element, functioning as a genetic data register. Flipping the register converts the flanking sites (triangle). The system is now in state 1 (S1). Alternatively a reset signal drives integrase and excisionase expression and restores register orientation and the flanking sites. The system is in its other state (S0). The register comprises a promoter, which is driving state dependent, strand-specific transcription of either red or green fluorescent protein, the two possible outputs of the system.

Several biological storage devices have been engineered [41] [42] [45] [159]. Some feedback motifs in natural systems exhibit memory such as mutual inhibition and auto regulatory positive feedback [41]. One synthetic example is a modular memory device that has been built *in vivo*, based on a transcriptionally controlled network, containing such an auto regulatory positive feedback (Figure 6A) [41].

Another study engineered an integrated logic and memory device, where the memory raised from the ability of recombinases to ‘write’ information in DNA [45].

One of the major goals in the field of biocomputing is to engineer *in vivo* a general form of state machine. This requires the ability to erase a symbol from the band of the machine and write a new symbol, thus to reversibly write information. Bonnet et al. built *in vivo* a rewritable recombinase addressable data module that stored data within a DNA sequence (Figure 6B) [42].

### Busses - wires

Wires in silicon microprocessors are made from solid state metals, whereas wires in biocomputers, in the systems engineered so far, consist of signaling molecules, such as regulatory proteins. This has been theoretically proposed by Sugita, as we have already discussed, and later executed in many systems. The quorum sensing system we mentioned above is one of many examples [112]. In this concrete case are the quorum sensing molecules are used as wires between different logic gates (Figure 4D).

## Potential applications of biological computers

Biological computers possess some distinct advantages over silicon computers [17] [18] [39]. These systems can self-assemble and self-reproduce, which might provide some economic advantages. Moreover, cells can be engineered to sense and respond to environmental signals, even under extreme conditions such as high temperature, high pressure, radioactivity or toxic chemicals. Biological systems have the ability to adapt to new information from a changed environment.

The ultimate goals of biocomputing are the monitoring and control of biological systems [18].

### Monitoring of biological systems

Biological systems need to be monitored in respect to disease diagnostic, to drug screening, to understand experimental systems, and to observe the environment [18].

In line with this, a biocomputer has been utilized to detect multiple disease indicators, such as mRNA of disease-related genes associated with small-cell lung cancer and prostate cancer [160] [161]. Moreover, they can be used in experimental models, such as conditional transgenes or inducible expression systems [162]. Environmental monitoring is another interesting application. A cell based biosensor using logic gates has been used to detect arsenic, mercury and copper ion levels [163].

### Control of biological systems

Biocomputers can potentially be used to control development, cell differentiation and re-programming, as all these processes depend on gene regulatory networks [18] [164]. Another application area is tissue engineering and tissue regeneration [165]. Metabolic engineering has the potential to produce from simple, inexpensive starting materials a large number of chemicals that are currently derived from nonrenewable resources or limited natural resources [166]. The metabolic flux can potentially be controlled by a biocomputer [120]. Interesting might also be to control the immune system by a biocomputer, e.g. in transplantation medicine [167]. An important application area is the control of malign growth. Some interesting experiments with logic based biological devices have been executed to detect cancer cells (e.g. small-cell lung cancer, prostate cancer, HeLa cells), and to induce selective apoptosis of these cells [77] [97] [160]. Furthermore, biocomputers can be used to engineer context-dependent programmable drugs [161] [125]. A biocomputer with a context-sensing mechanism, which can simultaneously sense different types of molecules, has been engineered [161]. In the future it might be used to detect a broad range of molecular disease symptoms, and react with the release of a drug molecule suitable for the treatment of the specific condition. In line with this concept a programmable NOR-based device has been developed capable of differentiating between prokaryotic cell strains based on their unique expression profile [125].

## Summary and outlook

Two decades have passed since the landmark paper of Adelman [35]. A major game changer has been the advance of synthetic biology, with novel concepts for bioengineering strongly based on systems theory. This led to trials for identifying, characterizing and standardizing biological parts useful for a general purpose computer. Major advances have been made in areas such as engineering of switches and logic gates, letting the dream of engineering a general Turing machine come close to reality. This dream is finally about our human superiority and rule over nature, making biocomputers one of the really exiting challenges in contemporary science, both in respect to engineering and ethics. We still face a couple of challenges before we will see biocomputers in our daily environment.

Novel concepts for Turing machines have been suggested, such as a deoxyribozyme based molecular walker, as this kind of machines have the ability to read and transform secondary cues [168]. However, the general Turing machine requires the ability of erasing and writing of symbols. Recently, major advantages have been made in respect to genome wide codon replacement *in vivo* by applying multiplex automated genome engineering technology [169]. This technology provides novel opportunities to implement a general Turing paradigm.

On this road we need to clarify whether the digital paradigm is in fact the best approach to molecular computing. As we have seen, the values of biological signals are typically analog, we need to explore, if analog computing might be an alternative road to explore. In any case, we need to engineer signals, both as input and output with well-defined stable concentrations, thus do not fluctuate, and stable circuits [170] [171] [172] [107]. If we wish to use Boolean logic we need to be able to group signals in low expressed and high expressed. The engineering design of the logic gate based on the transcriptor, as we discussed above, mark the advances that have been made towards digitalization of signals and the engineering of clearcut thresholds [134].

Moreover, the engineering of standardized reusable modules has been a major objective of synthetic biology. Signals are physically separated in microelectronics, contributing to standardization. Many biological devices, engineered so far, lack this signal separation, thus limiting the engineering of standardized, reusable modules [173]. Limitations towards this goal are due to circuit unpredictability, incompatible parts or random fluctuations. Moreover, wiring multiple logic gates is often difficult to implement reliably within mono cellular systems, as connections need to be implemented by a different molecule. One could potentially avoid this by using multiple cells in biological computer following the distributed computing paradigm of silicon computer, where a distributed system consists of multiple computers that communicate through a computer network [112] [143]. These multi cellular systems, as we discussed above, allows the output signal to be distributed among different cell types, which can be combined in multiple, reusable and scalable ways. Regot et al. demonstrated in yeast that these systems can reduces wiring constraints, which allowed the building of more complex synthetic devices, as they were able to implement many logic functions by using just a few engineered cells. [143]. In any case, we are still far from a general purpose computer, as also these kinds of systems will still be engineered with specific functions in mind.

Another area of focus might be processing speed, in general a critical factor for all forms of computation. Systems running on biological gates are relatively slow compared with silicon computers. The activation time both of biological logic devices systems ranges from seconds, as the CID system, to days as some transactivators and RNA aptamer [101]. In between do we have cell free logic devices which act in a time scale of minutes (protein enzyme, ribozyme) to hours (e.g. deoxyribozyme and branch migration). The cell based logic devices mainly have a typical activation time in the range of hours (e.g. miRNA, network plasmids, riboswitch, RNA aptamer, ribozyme, assembled RNA, intercellular networks, amber suppressor tRNA). The invention of other very rapid acting systems such as the CID system might be desirable. One might be able to take advantage of the fact that many cellular functions happen in parallel. Thus parallel computing paradigm might provide an interesting engineering paradigm. Moreover, optimization of individual components will increase processing speed [174].

Silicon computers have been a fruitful inspiration for the engineering of computing systems from biological materials. These engineered biological computers have some advantages over the silicon counterpart, as they can potentially self-organize and self-replicate. This has the potential to reduce engineering costs and efforts. However, the overall capabilities of today's artificial engineered biological computers are still premature in many aspects in comparison to the silicon based one. We have already discussed some of the technical reasons behind this, such as the limitations for building complex systems. We have seen, that today's logic gates can only be concentrated for up to order 10 processing steps [144]. We have discussed the problems of long processing time. The logic problems solved so far by biological computers are impressive, but also demonstrate the inferiority of such systems in comparison with their silicon counterparts, as they are still of relatively simple nature [35] [47] [86] [133] [147]. These problems are both due to the novelty of the field, but also, as we have seen, to system specific properties of the biological matter. As discussed, biochemical reactions have by nature often long reaction times [174]. The input and output signals are of analog and not digital nature [18]. Biochemical reactions are often in solution and not in all cases compartmentalized, which results, as discussed, in the lack of signal separation [173]. Novel compartmentalization concepts, organizing signal transduction by eg binding mediators to a scaffold, might further contribute to signal separation [118]. Although some solutions have been discussed above, these kinds of inert material properties might define the natural limitations for the engineering of biological computers. One might consider, a change in the computing paradigm applied, in order to engineer more in coherence with these material properties. The analog computer paradigm, which uses continuous values, might be interesting in this respect. Daniel et al have recently published a paper exploring analog computing in living cells [175]. They demonstrate that synthetic analog gene circuits can be engineered to execute sophistical computational functions in living cells. Moreover, further improvement might be possible to advancements in biological engineering. Standardized parts are, as discussed, the fundament, further engineering can build on [30] [31] [32]. Much of the work necessary is in line with standard quality insurance in biological experiments such as system stability and consistency under different conditions, system quantification, and identification of system imperfections [18]. Examples of such experimental problems are: systems might be unstable due to transient transfections. Moreover, cell populations might be not homogenous due to heterogeneity of gene copies, rate constants and stochastic effects. Furthermore, system measurements are potentially difficult in respect to measuring intracellular input levels. Once experimental advances are made towards standardized and well defined parts, one of the major next engineering steps will be to combine the different units of the biological microprocessor to one complex system. A challenge will be the spatial organization of such a complex system. Novel artificial scaffold systems might be necessary to develop for this purpose. Efficient manufacture methods might also be required. The emerging field of 3-D printing might provide novel ways for system engineering. Further advancements in engineering of biological control units might be necessary for powerful integrated systems. Altogether, this will push biological systems closer to the level of complexity and problem solving power of silicon computers. Such an integrated system will have much more computing power and advances the problem solving capability. Evidence for the potential of the potential computing power of a biological system is provided by the capabilities of nature's most powerful biological computer, the human brain.

Novel areas for development are on the horizon. Hybrids of electronic semiconductor and biological machines might be interesting to explore; playing on the initial discussed feedback loop between biology inspired engineering and engineering inspired biology [176] [177]. Some interesting research is going on in this area both in academic labs and in industry. Several promising biocomposites have been developed, such as cells treated with silicic acid; DNA as a mediator that arranges fullerenes, golden particles and DNA-templated nanowire formation; and DNA metamaterials and hydrogels with memory [178] [179] [180] [181] [182] [183]. Another interesting device under development is IBM's DNA transistor [184]. This system controls DNA translocation through the nanopore. It is composed of a metal/dielectric/metal/dielectric/metal multilayer nano-structure built into the membrane that contains the nanopore. The function of this system is based on the interaction of discrete charges along the backbone of a DNA molecule with the modulated electric field to trap DNA in the nanopore with single-base resolution. DNA might be moved through the nanopore at a rate of one nucleotide per cycle. This could lead among other to a nanopore-based reading device.

Finally, as the young field of synthetic biology and systems biology most likely will further advance in the years to come, so will biocomputing. A biological microprocessor, an implication of a general Turing machine is on the horizon.
